# In the carotid body, galanin is a signal for neurogenesis in young, and for neurodegeneration in the old and in drug-addicted subjects

**DOI:** 10.3389/fphys.2014.00427

**Published:** 2014-10-31

**Authors:** Andrea Mazzatenta, Guya D. Marconi, Susi Zara, Amelia Cataldi, Andrea Porzionato, Camillo Di Giulio

**Affiliations:** ^1^Physiology and Physiopathology Section, Department of Neurosciences, Imaging and Clinical Science, University of Chieti–PescaraChieti, Italy; ^2^Department of Drug Sciences, University of Chieti–PescaraChieti, Italy; ^3^Department of Human Anatomy and Physiology, University of PaduaPadua, Italy

**Keywords:** galanin, human carotid body, neuronal-like cell, chemoreception, hypoxia, neurogenesis, drug addiction, aging

## Abstract

The carotid body is a highly specialized chemoreceptive structure for the detection of and reaction to hypoxia, through induction of an increase in hypoxia inducible factor. As tissue hypoxia increases with aging and can have dramatic effects in respiratory depression induced by drug addiction, we investigated the carotid body in young and old healthy subjects in comparison with drug-addicted subjects, including the expression of the neurotransmitter galanin. Galanin expression was recently reported for neuronal-like cells of the human carotid body, and it is implicated in several functions in neurons. In particular, this includes the regulation of differentiation of neural stem cells, and participation in the development and plasticity of the nervous system. Using immunohistochemistry detection, we demonstrate that galanin expression in the human carotid body in healthy older subjects and drug-addicted subjects is significantly reduced in comparison with healthy young subjects. This demonstrates not only the effects of normal aging and senescence, but also in the drug-addicted subjects, this appears to be due to a disorganization of the chemo-sensory region. With both aging and drug addiction, this results in a physiological reduction in neuronal-like cells, coupled with interlobular and intralobular increases in connective tissue fibers. Consequently, in both aging and drug addiction, this reduction of neuronal-like cells and the regeneration suggest that the carotid body is losing its sensory capabilities, with the transmission of chemoreceptive signals dramatically and vitally reduced. The level of galanin expression would thus provide a signal for neurogenesis in young subjects, and for neurodegeneration in older and drug-addicted subjects.

## Introduction

In humans, the pleiotropic 30-amino-acid neuropeptide galanin is widely distributed in the central nervous system, where it is biologically active and participates in the modulation of several ascending neurotransmitter systems, including cholinergic, noradrenergic, and serotoninergic pathways (Tatemoto et al., 1983; Crawley et al., 2002). Three galanin receptors have been identified (GalR1-3), and these signal through G-protein-coupled mechanisms in tissue-specific and cell-specific manners, to modulate a wide array of homeostatic and pathological processes (Counts et al., 2003). Galanin acts as neurotrophic/neuroprotective factor for several neuronal populations, and it is involved in the plasticity of the nervous system. Furthermore, galanin administration results in up-regulation of genes involved in pro-survival/pro-neuronal signaling pathways, and increases the number of neurons arising from differentiation of olfactory sensory neuron progenitors (Cordeo-Llana et al., 2014). Treatment of wild-type and GAL knock-out neural stem cells with galanin and the GalR2-specific agonist Gal2-11 under differentiation conditions significantly promotes neuritogenesis, which is inhibited by the galanin antagonist M35 (Ma et al., 2008). Galanin thus regulates differentiating neural stem cells, and in this way it participates in the development and plasticity of the nervous system.

Recently, Di Giulio et al. (2014) reported on the selective expression of galanin in neuronal-like cells in the human carotid body. In previous studies, galanin has been described in the animal carotid body (Kameda, 1989; Ichikawa and Helke, 1993; Finley et al., 1995). Furthermore, in a study on the presence and localization of the three galanin receptor subtypes (at the mRNA and protein levels), GalR1 and GalR2 were identified in neuronal-like cells, but not in sustentacular cells, while GalR3 was negative for the whole of the carotid body (Porzionato et al., 2010).

The carotid body is a well-defined chemoreceptive anatomical structure with contiguity of function with the carotid arterial bifurcation. Its specialized physiological role is as an arterial chemoreceptor, to modulate the ventilatory volume and frequency in response to hypoxia, hypercapnia, and acidosis. The carotid body has a lobular organization, with the lobes separated by thin connective septa. The cellular component includes neuronal-like, or Type I, cells, and sustentacular, or Type II, cells (Verna, 1979; Pallot, 1987). The neuronal-like cells are considered to be chemoreceptor units (Prabhakar, 2000, 2006; López-Barneo et al., 2008) that can release several neurotransmitters and neuromodulators in response to stimulating conditions (Iturriaga and Alcayaga, 2004; Nurse, 2005; Shirahata et al., 2007; Porzionato et al., 2008). This, in turn, elicits nervous impulses that are conveyed through glosso-pharyngeal afferent fibers that arise from the petrosal ganglion (Iturriaga et al., 2007). The sustentacular cells are glial-like cells that express astrocytic markers and have a supportive role (Pallot, 1987), although when exposed to prolonged hypoxia, they have been described as having a role in the production of stem-cell precursors for neuronal-like cells (Pardal et al., 2007).

In aging and, in particular, in a state of drug addiction, the carotid body undergoes several morphological, physiological, and biochemical modifications (Porzionato et al., 2005; Zara et al., 2013). Typically, there are changes in the cell populations, such as an increase in the connective tissue fiber compartment, which results in a reduction in the sensory tissue, and represents a sign of tissue aging. In drug-addicted subjects, early tissue aging has been coupled to episodes of respiratory depression and arteriosclerosis of the glomic arteries (Di Giulio et al., 2003; Porzionato et al., 2005; Zara et al., 2013).

In the present study, we investigated the expression of galanin in the human carotid body in young and old healthy subjects, in comparison with drug-addicted subjects. Our data indicate that galanin provides a signal for neurogenesis in young subjects, and for neurodegeneration in older subjects and in those under the pathological conditions of drug addiction.

## Materials and methods

In the present study were selected human carotid bodies (*n* = 15) that were collected from 12- to 72-h postmortem subjects without chronic pulmonary or cardiovascular disease. Exclusion criteria as cardiac hypertrophy or previous myocardial infarction were also excluded at autopsy examination. Pathological carotid body specimens (*n* = 8; males; mean age, 27 ± 3.5 years) were collected from subjects with a history of drug addiction and with drug taking as the cause of death. Toxicological investigations for a group of drugs, including cocaine, methadone, amphetamines, benzodiazepines, cannabis, and alcohol, were performed on urine samples and venous blood samples, across concentration ranges of 0.5–103.2 mg/ml and 0.5–31.1 mg/ml, respectively. The control carotid body specimens were collected from young males (*n* = 3; mean age, 30 ± 3.5 years) and older males (*n* = 4; mean age, 70 ± 5.5 years) who had died by accidental trauma. The urine and venous blood samples of these controls were negative in the toxicological investigations. The study received ethical approval from the local Research Board.

The specimens were fixed in neutral 10% formalin, embedded in paraffin wax, and sectioned (5 μm), followed by histological staining with Mallory trichrome (Bio Optica; Milan, Italy). The mouse monoclonal anti-galanin antibody (H-11: sc166431; Santa Cruz Biotechnology; CA, USA) and anti-hypoxia-inducible factor (HIF) antibody (H1α 67, sc-53546; Santa Cruz Biotechnology; CA, USA) and developing kits (UltraVision LP Detection System HRP Polymer & DAB Plus Chromogen, Lab Vision Thermo Scientific; CA, USA) were used for the immunohistochemistry. For light microscopy and the data acquisition system, a Leica DM 4000 microscope was used, which was equipped with a Leica DFC 320 digital acquisition system (Leica Cambridge Ltd.; Cambridge, UK). QWin Plus 3.5 software (Leica Cambridge Ltd.; Cambridge, UK) was used to digitize the images and to compute the areas positive for the antibodies. Commercial software (SPSS and Origin) were used for the data and statistical analyses (One-Way ANOVA, with α level set at 0.001, or as specified).

Tissue degeneration and alteration detected by Mallory trichrome histological staining were considered as exclusion criteria. To detect any influence of the death-to-autopsy interval carotid body volume was assessed by Cavalieri's method. Further, the ratio between the areas occupied by connective tissue and parenchyma, such as intralobular connective tissue, and the area exhibited by the whole organ in five sections per subjects was taken as an estimate of the corresponding volume fractions, statistical analysis of the linear correlations was carried out under each condition, with an α level set at 0.05 (for detail in the method see Porzionato et al., 2005).

## Results

**Figure 1:**
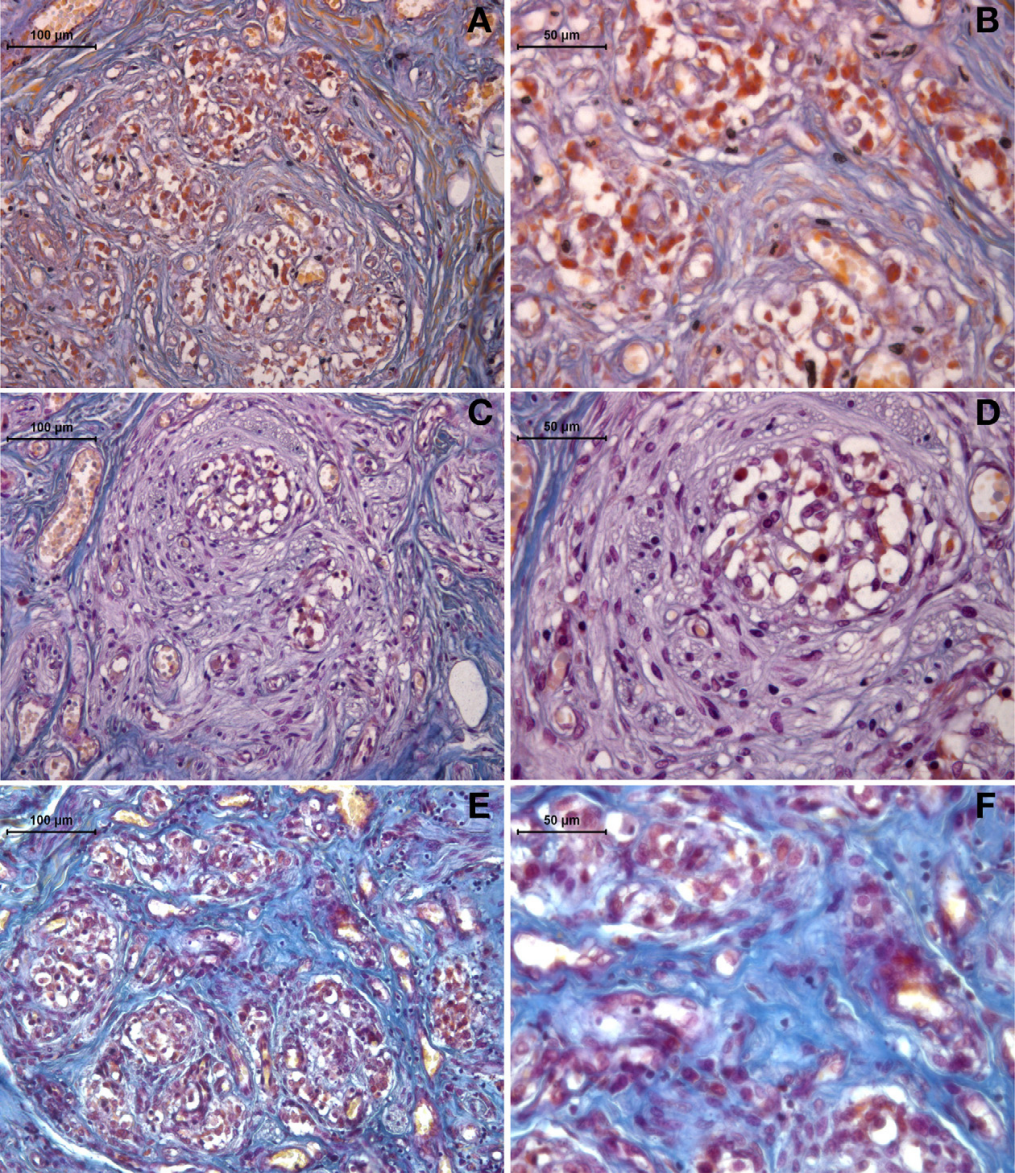
**Representative Mallory trichrome staining of human carotid body sections in young (A,B) and old (C,D) healthy subjects, compared to drug-addicted subjects (E,F)**. With the drug addiction, note the pathological disorganization of the sensory region and the increase in connective tissue fibers (blue), as interlobular (b) and intralobular (c).

**Figure 2:**
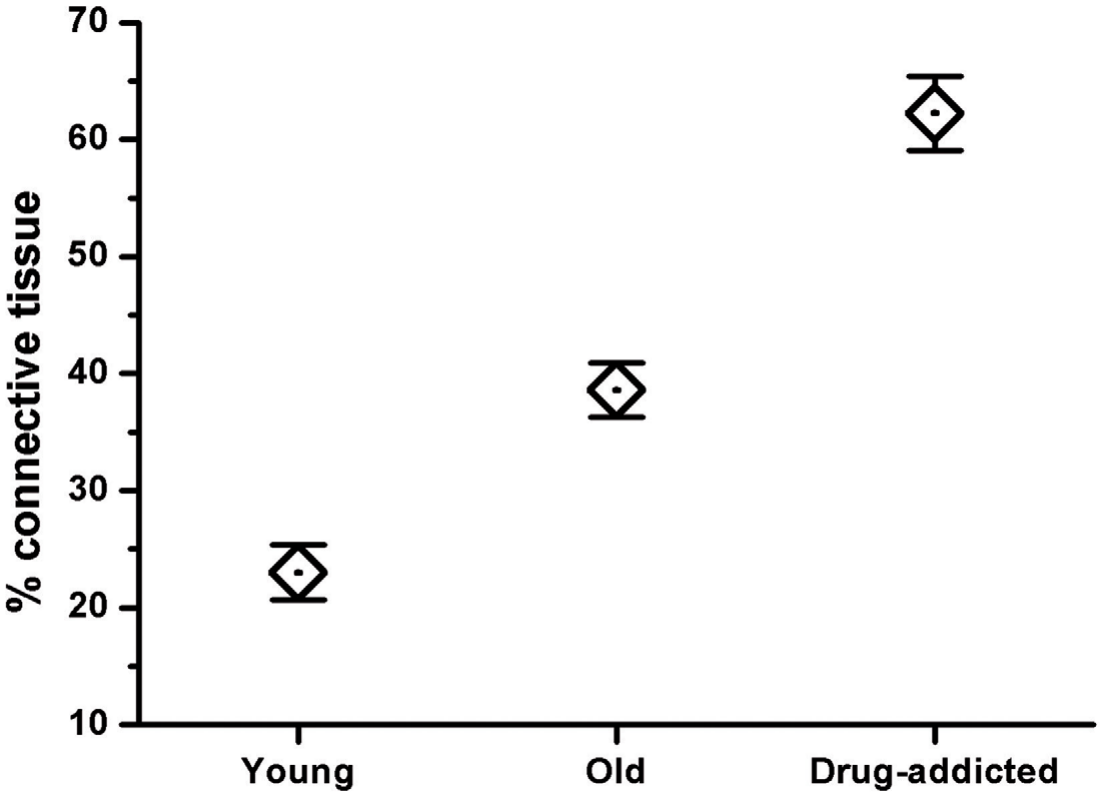
**Morphometric analysis of the connective tissue fibers in human carotid body sections in healthy (young and old) and drug-addicted subjects, performed on Mallory trichrome staining**. The One-Way ANOVA demonstrates a significant increase in the connective tissue fibers through the aging process and with drug addiction (*p* < 0.001).

The Mallory trichrome staining of the human carotid body sections in the young and old healthy controls (Figure 1) showed tissue senescence related to aging. In the sections from the drug-addicted subjects, there was apparent pathological disorganization of the sensory areas and increases in both the interlobular and intralobular connective tissue fibers (Figure 2). The One-Way ANOVA defined a significant increase in connective tissue fibers in the older and drug-addicted subjects [*F*_(2, 33)_ = 56.3; *p* < 0.001], with *post-hoc* One-Way ANOVA showing significant increases in the connective tissue fiber with aging [young vs. old: *F*_(1, 24)_ = 22.0; *p* < 0.001], and between the young healthy subjects and those who were drug-addicted [*F*_(1, 22)_ = 102.6; *p* < 0.001].

**Figure 3:**
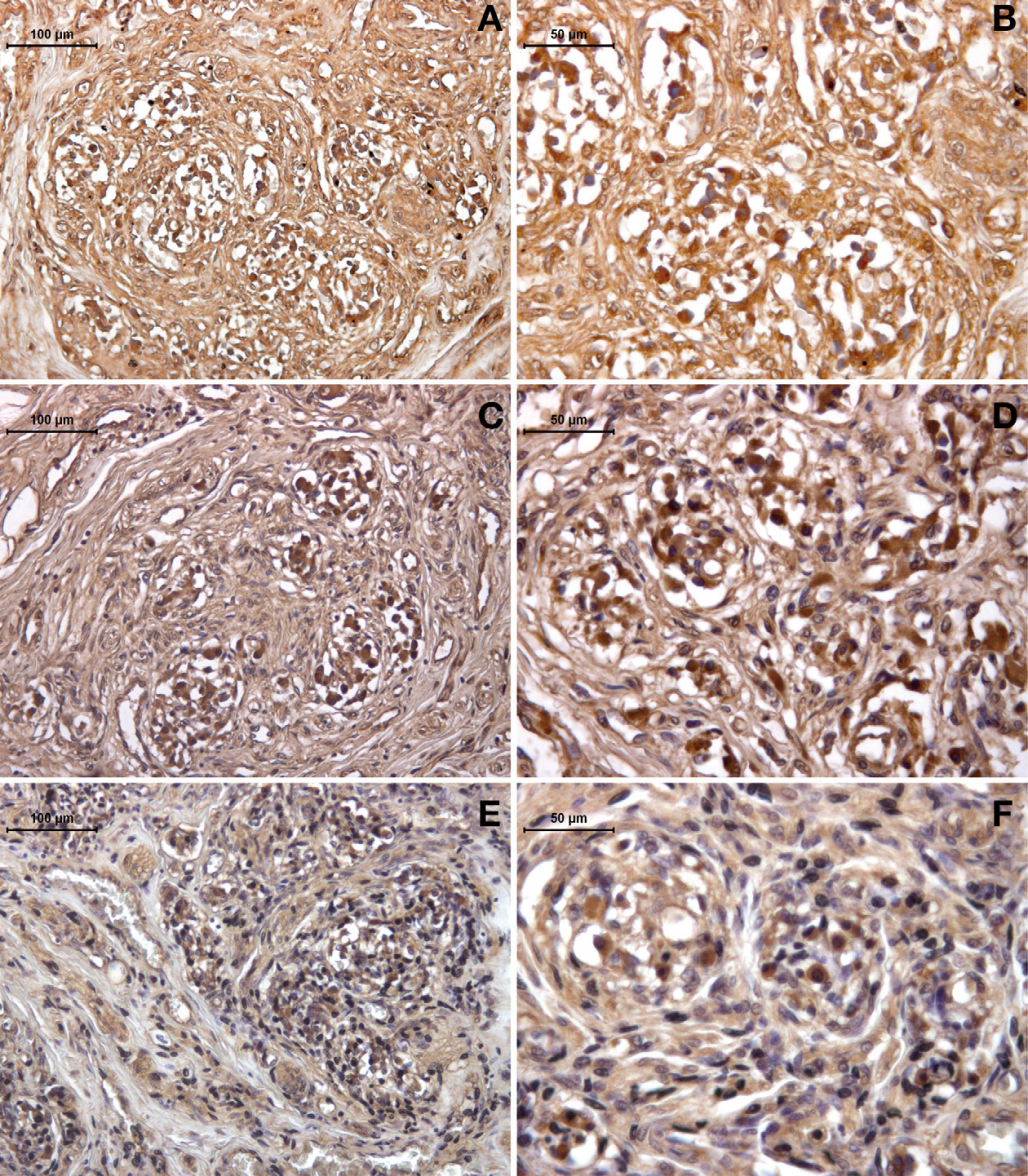
**Immunohistochemistry detection of galanin expression in human carotid body, in young (A,B) and old (C,D) healthy subjects, compared to drug-addicted subjects (E,F)**. With the drug addiction, note the scarce labeling that is higher than for the old healthy subjects, which shows a dramatic effect of the disorganization in the sensory region with the reduction of the neuronal-like cells and increases in the connective tissue fibers, seen as interlobular (left panel) and intralobular at higher magnification (right panel).

The expression of galanin was revealed by immunohistochemistry in the sections from the healthy (young and old) and drug-addicted subjects. In the young healthy subjects, positive galanin labeling was restricted to the sensory areas at the level of the neuronal-like cells, while in the older healthy subjects, the labeling was decreased in this area; there was also a further dramatic reduction in galanin expression in the tissues from the drug-addicted subjects (Figure 3). One-Way ANOVA showed significant decreases in galanin labeling with aging [young vs. old: *F*_(1, 23)_ = 12.5; *p* < 0.001] and in the young healthy vs. drug-addicted subjects [*F*_(1, 23)_ = 21; *p* < 0.001], as also seen between all of the groups [*F*_(2, 37)_ = 14.7; *p* < 0.001].

**Figure 4:**
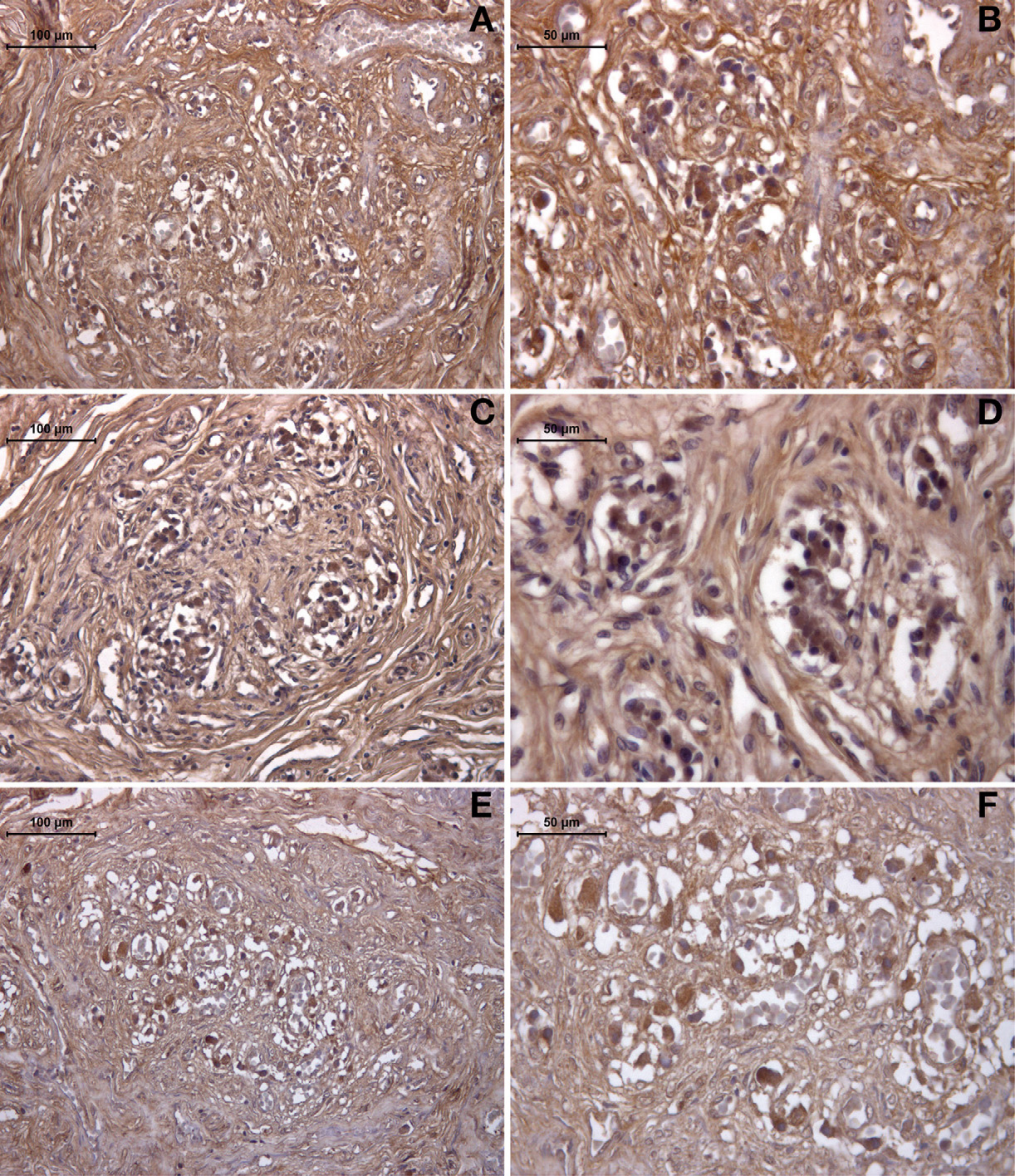
**Immunohistochemistry detection of HIF expression in the human carotid body, in young (A,B) and old (C,D) healthy subjects, compared to drug addicted subjects (E,F)**.

The expression of HIF was also revealed by immunohistochemistry in the healthy (young and old) and drug-addicted subjects (Figure 4). One-Way ANOVA showed significant increases in HIF labeling with aging [young vs. old: *F*_(1, 9)_ = 10.6; *p* < 0.001], and in the young healthy vs. drug-addicted subjects [*F*_(1, 14)_ = 4.9; *p* < 0.05], as also between all of the groups [*F*_(2, 21)_ = 4.25; *p* < 0.05].

**Figure 5:**
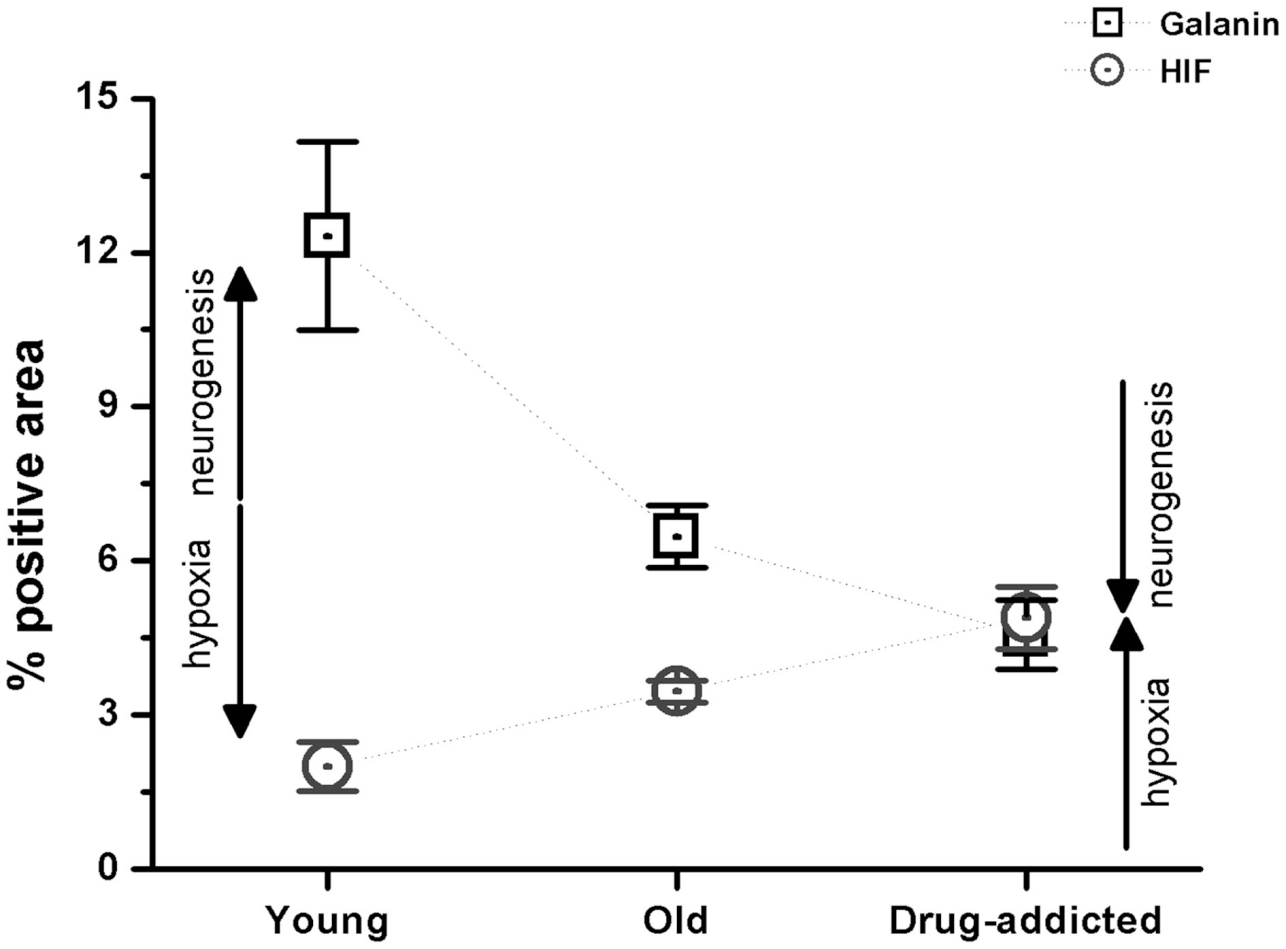
**Morphometric analysis of the positive areas for galanin and HIF in the human carotid body sections from the healthy young and old subjects, compared to the drug-addicted subjects, performed on anti-galanin immunohistochemistry labeling**. One-Way ANOVA demonstrates a significant decrease in galanin expression on aging, and higher levels in drug-addicted subjects (*p* < 0.001); conversely, there is a significant increase in HIF expression upon aging, that is greater for the drug-addicted subjects.

The effects of neurogenesis and hypoxia were also compared between the healthy (young and old) and drug-addicted subjects, as for galanin and HIF expression (Figure 5).

There were no statistically significant differences in the mean death-to-autopsy intervals between these healthy (young and old) and drug-addicted subjects. Furthermore, there were no statistically significant correlations seen between the death-to-autopsy interval on each parameter investigated (*p* < 0.05).

## Discussion

Although increases in connective tissue fibers are not exclusive to the aging process, its occurrence in this young population of drug-addicted subjects suggests activation of an early aging mechanism in the carotid body that would be due to the hypoxia effects of drug addiction (Porzionato et al., 2005; Zara et al., 2013). We have already shown that HIF expression increases with the aging process and in drug addiction (Zara et al., 2013), with the present data further confirming these previous data.

This early-aging phenomenon also appears to be also coupled with a selective reduction in the neuronal-like cells, which would also represent a specific character of this hypoxia effect. Furthermore, the most important evidence of the disorganization of the sensory region of the carotid body in these drug-addicted subjects was the significant reduction in galanin expression and the increase in HIF expression. The significance of these findings is in the line with a recent report where a loss of galanin led to a marked decrease in the rate of adult neurogenesis and a reduction in the number of newly generated cells in the olfactory bulb (Cordeo-Llana et al., 2014).

The novelty of the present study is that it provides intriguing aspects related to the putative function of galanin in the carotid body. Galanin levels are potentially related to neuronal differentiation, such as was seen for neuroregeneration of olfactory sensory neurons (Cordeo-Llana et al., 2014). Galanin was expressed selectively in differentiating neuronal-like cells, and this is in line with what we have indirectly shown in the present study for the carotid body. This loss of galanin expression following aging and drug addiction, thus indicates here a reduction in regenerating neuronal-like cells, which in turn might suggest that the carotid body is losing its sensory capabilities. This is further supported by the increased levels of immunostaining for HIF, a known response to hypoxia (Semenza, 2000), and related to the aging process and to drug addiction (Zara et al., 2013; present study). As a consequence, the transmission of chemoreceptive signals appears to be dramatically and vitally reduced.

In conclusion, the consequences of aging and drug addiction seen here indicate severe cardio-respiratory impairment with an accelerated aging process. Our findings thus provide further evidence of the role of galanin as a modulator of neural stem cell function, and reinforce the importance of galanin for brain plasticity and repair.

### Conflict of interest statement

The authors declare that the research was conducted in the absence of any commercial or financial relationships that could be construed as a potential conflict of interest.
